# Oculocutaneous albinism: the neurological, behavioral, and neuro-ophthalmological perspective

**DOI:** 10.1007/s00431-023-04938-w

**Published:** 2023-04-03

**Authors:** Jessica Galli, Erika Loi, Laura Dusi, Nadia Pasini, Andrea Rossi, Vera Scaglioni, Lucia Mauri, Elisa Fazzi

**Affiliations:** 1 Department of Clinical and Experimental Sciences, University of Brescia, Brescia, Italy; 2 Unit of Child Neurology and Psychiatry, ASST Spedali Civili of Brescia, Brescia, Italy; 3 Department of Molecular and Translational Medicine, University of Brescia, Brescia, Italy; 4 Department of Neurological and Vision Sciences, ASST Spedali Civili of Brescia, Brescia, Italy; 5 Medical Genetics Unit, Department of Laboratory Medicine, ASST Grande Ospedale Metropolitano Niguarda, Milan, Italy

**Keywords:** Albinism, Children, Neurodevelopment, Vision, Intelligence quotient

## Abstract

Oculocutaneous albinism (OCA) is a group of rare, genetic disorders caused by absent/reduced melanin biosynthesis. The aim of this study was to explore the neurovisual, cognitive, adaptive, and behavioral profile of children affected by OCA, also evaluating any possible effect of the visual acuity deficit on the clinical profile and genotype–phenotype correlations. Eighteen children (9 males, mean age 84 months ± 41; range 18–181 months) with a molecular confirmed diagnosis of OCA were enrolled in the study. We collected data on clinical history, neurodevelopmental profile, neurological and neurovisual examination, and cognitive, adaptive, and emotional/behavioral functioning. A global neurodevelopmental impairment was detected in 56% of the children, without evolving into an intellectual disability. All the patients showed signs and symptoms of visual impairment. Low adaptive functioning was observed in 3 cases (17%). A risk for internalizing behavioral problems was documented in 6 cases (33%), for externalizing problems in 2 (11%), and for both in 5 (28%). Twelve children (67%) showed one or more autistic-like features. Correlation analyses revealed significant associations between the visual acuity level and performance intelligence quotient (*p* = 0.001), processing speed index (*p* = 0.021), Vineland total score (*p* = 0.020), Vineland communication (*p* = 0.020), and socialization (*p* = 0.037) domains. No significant correlations were found between genotype and phenotype.

*Conclusion*: Children with OCA may present a global neurodevelopmental delay that seems to improve with age and emotional/behavioral difficulties, along with the well-known visual impairment. An early neuropsychiatric evaluation and habilitative training are recommended to improve vision-related performance, neurodevelopment, and any psychological difficulties.

**Table:** 

**What is Known:** *• Children with oculocutaneous albinism show dermatological and ophthalmological problems*. *• An early visual impairment may have negative implications on motor, emotional, and cognitive processes that would allow the child to organize his or her experiences*.	
**What is New:** *• In addition to a variable combination of ocular signs and symptoms, children with oculocutaneous albinism may present an early neurodevelopmental delay and emotional/behavioral difficulties*. *• An early visual treatment is recommended to improve vision-related performance, neurodevelopment, and any psychological difficulties*.

**Supplementary Information:**

The online version contains supplementary material available at 10.1007/s00431-023-04938-w.

## Introduction

Oculocutaneous albinism (OCA) is a rare, autosomal recessive disorder caused by the complete absence or reduction of biosynthesis of melanin in melanocytes. It affects people globally, with an overall prevalence of approximately 1 in 20,000 [1], with different rates across geographic regions and ethnic groups [2].

Individuals affected by OCA present a normal number of melanocytes in the epidermis and follicles, but they totally or partially lack of the melanin pigment [3]. Currently, eight forms have been identified (from OCA1 to OCA8) with a highly variable phenotype [1, 4, 5]. Ocular abnormalities, such as hypopigmentation of iris and retina, foveal hypoplasia, and atypical decussation at the optic chiasm, also characterize the disorder, resulting in a wide spectrum of visual signs and symptoms, such as reduced visual acuity, impaired stereopsis, strabismus, nystagmus, iris translucency, and photophobia [6].

Several alterations in genes encoding proteins involved in the melanin biosynthesis pathway have been identified: specifically, the *TYR* gene (OCA1, both A and B type), *OCA2* or* P* gene (OCA2), *TYRP1* (OCA3) [7], *SLC45A2* (OCA4) [4], *SLC24A5* (OCA6) [8], *LRMDA* (OCA7) [9], and DCT (OCA8) [10]; the genetic mutation for the OCA5 subtype has not yet been identified but mapped to chromosome 4q24 [11]. Finally, OCA can also occur in several syndromic disorders, such as the Hermansky–Pudlak syndrome [12] and the Chediak–Higashi syndrome [13].

To date, neurodevelopmental outcomes in subjects with OCA are poorly investigated, and the literature is limited to few studies [14–18] and case reports [19–21]. Kutzbach and colleagues [14, 15] reported the presence of attention-deficit/hyperactivity disorder in 6.8% of adults and in 21.7% of children with OCA and of autism spectrum disorders (ASDs) in 3.8% of children; no reading disorders were found in the 44 children evaluated. As regards cognitive profile, normal quotient has been reported in albino subjects [16–18]. Finally, in a recent study by our group on children with ocular albinism (OA) [22], we documented neurodevelopmental problems (such as developmental delay, impaired cognitive profile, language disorder, and autistic-like features) above all in children with a genetically confirmed OA diagnosis. The association between OCA and neurodevelopmental disorders could be explained by genetic factors (mutation of the *GABRA5* gene, candidate for autism, and located near to the *OCA2* gene) [20] and brain connectivity dysfunctions (misdirection in central nervous system networks other than the visual pathways) [14, 21, 22].

Despite some descriptive data documenting the cognitive level and academic skills of albino subjects, no studies, to our knowledge, provide a comprehensive description of their clinical profile, collecting and relating different developmental areas such as neurovisual, cognitive, adaptive, and behavioral features. Given these premises, the aim of the present study was (1) to explore these neurodevelopmental areas that characterize children affected by OCA; (2) to evaluate any possible effect of the visual acuity deficit on cognitive, adaptive, and behavioral functions; and (3) to explore any possible genotype–phenotype correlations.

## Methods

We collected and analyzed demographic, genetic, and clinical data of 18 children (9 males, mean age of 84 months, SD 41; range 18–181 months), referred to our Neuro-ophthalmological Tertiary Center, Child Neurology and Psychiatry Unit, ASST Spedali Civili of Brescia between January 2021 and July 2022. Inclusion criteria were the diagnosis of OCA, confirmed by genetic analysis, and age from birth to 18 years. Exclusion criteria were the presence of syndromic forms of albinism or ocular albinism. None of the eligible subjects declined to participate.

Data on clinical history (pregnancy, delivery, birth weight, and neurodevelopment milestones) and on neurodevelopmental profile, evaluated using the Griffiths Mental Developmental Scales–Revised (GMDS-R) [23], were collected from medical charts. Furthermore, the enrolled children underwent neurological and neurovisual examination, cognitive evaluation, and adaptive and emotional/behavioral functioning assessments.

The neurovisual profile was assessed according to our protocol [24–26] that includes the evaluation of ophthalmological characteristics (refraction under cycloplegia, anterior segment, and ocular fundus); oculomotor functions as fixation (defined as “altered” when unstable or absent), smooth pursuit (“altered” when discontinuous or difficult to elicit/absent), saccadic movements (“altered” when dysmetric and/or with increased latency or absent), strabismus, ocular motility deficit, and abnormal eye movements; and basic visual functions (visual acuity, contrast sensitivity, visual field). Visual acuity was evaluated under maximum refractive correction with test suitable for patient’s age and cooperation using Teller Acuity Cards [27], Lea symbols or letter optotypes [28]. We defined visual acuity score as “normal” or “reduced” according to normative data [27, 29, 30]. Contrast sensitivity was evaluated using the Hiding Heidi Low Contrast “Face” Test and considered as “reduced” if > 2.5%, while the visual field was evaluated through the kinetic perimetry [31].

Cognitive level was evaluated using age-appropriate versions of Wechsler Preschool and Primary Scale of Intelligence III edition (WPPSI-III) [32] and Wechsler Scales of Intelligence for Children IV edition (WISC-IV) [33]. Full-scale intelligence quotient (FSIQ), verbal comprehension/verbal intelligence quotient (VCI/VIQ), perceptual reasoning/performance intelligence quotient (PRI/PIQ), working memory (WMI), and processing speed index (PSI) scores were collected. All the quotients are reported in standard scores (mean 100, SD 15) and defined as follows: more than −1 DS, normal; between −1 and −2 DS, borderline; and < −2 DS, delay.

Adaptive functioning was evaluated using the Vineland Adaptive Behavior Scales-II (VABS-II) [34], a questionnaire filled out by parents that covers four domains of adaptive behavior that is communication, socialization, daily living skills, and motor skills. Centile score <5 was considered as a cut-off for adaptive disorders.

Emotional and behavioral characteristics were assessed using the parent report Child Behavior Checklist (CBCL) 1½-5 [35, 36] or 6–18 [37] according to age. Composite scales (internalizing, externalizing, and total problems) and syndrome scales (CBCL 1½-5: emotionally reactive, anxious/depressed, somatic complaints, withdrawn, sleep problems, attention problems, and aggressive behavior; CBCL 6–18: anxious/depressed, withdrawn/depressed, somatic complaints, social problems, thought problems, attention problems, rule-breaking behavior, and aggressive behavior) are obtained from the item scores, categorized as “normal,” “borderline,” or “clinically significant” according to the tool kit software standards. Given the high frequency of autistic-like features in visually impaired children [38] and in children with ocular albinism [22], we performed the Checklist for Autism Spectrum Disorder (CASD) [39]. Scores of 15 or higher are within the autism range, scores of 10–14 in the autism spectrum range, and scores less than 10 characterize children with typical development or different diagnoses.

The study was conducted in accordance with the ethical guidelines set forth by the Declaration of Helsinki and was approved by the Ethical Committee of ASST Spedali Civili in Brescia, Italy (NP 5523). Written informed consent was obtained from parents/caregivers of the children.

## Statistical analysis

A descriptive analysis of the findings was performed. Qualitative variables were analyzed in terms of number and percentage, while quantitative data were reported as mean, standard deviation, and range. The Pearson or Spearman correlation analysis has been performed using SPSS software to evaluate the impact of the visual acuity deficit on cognitive level, adaptive functioning, and emotional/behavioral profile. Given limitations in genotype incidence, we were able to compare only two genotypes: OCA1 and OCA2. We applied a Fisher’s exact test to study the relationship between neurodevelopmental, cognitive, adaptive, and behavioral aspects and genetic defects. A *p* value below 0.05 was interpreted as statistically significant. When missing data were present due to the age of the children, we excluded from the analyses of the specific variable those subjects with missing data.

## Results

### Data collection

Out of the 18 subjects enrolled, 10 children (56%) had a *TYR* gene alteration (OCA1), 7 (38%) an *OCA2* gene deletion or mutation (OCA2) and 1 (6%) a *SLC45A2* gene deletion (OCA4).

From the medical chart revision, pregnancy was uneventful in 16 (89%) cases, while the mothers of 2 children (11%) suffered from gestational diabetes. Delivery was at term in 17 (94%) cases while a boy was born at 35 weeks of gestation. The mean birth weight was 3129.5 ± 426.0 g (range: 2540–4000 g). Perinatal was uneventful in all the sample.

Eight (44%) children appropriately acquired the gross (head control, sitting, walking) motor milestones, while 10 (56%) had different levels of delay: 5 (28%) presented with a delay in the head control, 5 (28%) in the sitting position, 4 (22%) in the walking, and 2 (11%) an overall delay. Specifically, the mean age of head control was 3 ± 1 months (range 2–6 months), sitting 7 ± 2 (range 5–12 months), and walking 16 ± 4 (range 10–24 months). Thumb finger was appropriately acquired in all the cases (mean age 12 ± 1 months, range 12–14 months). Data on communication and language skills were collected in 16 children; the remaining two were infants (aged between 18 and 21 months) and have not yet achieved the vocabulary expansion. Six (38%) children were *late talkers* because they produced fewer than 50 words or no word combinations at 24 months of age. Four of these eventually met their same-age peers in language performance, and therefore, they can be considered *late bloomers*; the remaining two *late talkers* received a diagnosis of language disorder at age six because they showed impairments in both receptive and expressive language skills on specific neuropsychological test. More in general, canonical babbling was acquired at 8 ± 1 months (range 7–12 months), 1st word at 16 ± 5 (range 10–27 months) and >50 words at 30 ± 10 months (range 24–60 months).

The developmental profile assessment evaluated in 16 children using GMDS-R (mean age at evaluation: 19 months ± 12, range 6–24) revealed normal scores on developmental quotient (DQ) in 7 cases (44%), borderline score in 5 (31%), and delayed score in 4 (25%). The remaining two children referred to the center in older ages and, therefore, did not undergo the GMDS-R assessment. The analysis of each domain revealed borderline/delay scores as follows: locomotor (3 borderline, 4 delay), personal–social (2 borderline, 6 delay), hearing–speech (2 borderline, 5 delay), eye–hand coordination (2 borderline, 7 delay), and performance (3 borderline, 5 delay).

### Direct assessment

Direct assessment was carried out at mean age of 84 months (SD 41; range 18–181 months). No abnormalities concerning cranial nerve, head circumference, muscle strength, tone and bulk, reflexes, and gait were detected.

From an ophthalmological perspective, all the 18 (100%) children presented refractive errors. Iris translucency and ocular fundus abnormalities characterized by hypopigmentation of retina and/or foveal hypoplasia were detected in 17 (94%) and 18 (100%) children, respectively. Eight children (44%) had photophobia and 16 (89%) an abnormal head position. As regards oculomotor functions, strabismus was observed in 14 (78%), nystagmus in 18 (100%), and alterations in fixation, smooth pursuit, and saccadic movements in 18 (100%), 17 (94%), and 13 (72%) children, respectively. The visual acuity was reduced in all the sample (100%), contrast sensitivity in 6 (33%), and visual field in 6 (33%). See Table 1.

**Table 1:** Neurovisual findings of the sample

**Sbj**	**Refr. errors**	**Ny**	**Strabismus**	**Fixation**	**Smooth pursuit**	**Saccades**	**Visual acuity**	**Contr Sens.**	**Visual field**
1	As, H	Pend	Exo	A	A	A	0.2	1.25	N
2	As, M	Jerky	No	A	A	A	0.06	2.5	N
3	As, M	Pend	Eso	A	A	A	0.05	1.25	N
4	As, M	Pend	Eso	A	A	A	0.1	1.25	A
5	As, H	Pend	Exo	A	A	N	0.1	1.25	A
6	As, M	Pend	Eso	A	A	A	0.1	5	A
7	As, H	Pend	Exo	A	A	A	0.15	1.25	N
8	As, H	Pend	Eso	A	A	A	0.1	1.25	A
9	As, M	Mix	Eso	A	A	A	0.1	1.25	N
10	As, M	Jerky	Exo	A	A	A	0.05	1.25	N
11	As, M	Mix	Exo	A	A	N	0.15	1.25	A
12	As, H	Pend	Exo	A	A	A	0.3	1.25	N
13	As, H	Jerky	Eso	A	N	A	0.2	1.25	N
14	As	Pend	No	A	A	N	3.1*	1.25	N
15	As	Mix	No	A	A	A	4.7*	5	N
16	As, M	Mix	No	A	A	A	4.7*	5	N
17	As, M	Pend	Eso	A	A	N	3.1*	5	A
18	As, M	Pend	No	A	A	N	0.9*	100	N

*A* altered, *As* astigmatism, *Contr. Sens.* contrast sensitivity, *Eso* esotropia, *Exo* exotropia, *H *hypermetropia, *M* myopia, *N* normal, *Ny* nystagmus, *Pend* pendular, *Refr. errors* refractive errors

*values are reported in cyc/deg

The cognitive evaluation was performed in 14 children (the remaining four subjects were too young for the WPPSI/WISC-IV assessment) and showed normal FSIQ in 11 children (79%), borderline score in 2 cases (14%), and deficient in 1 (7%). VIQ was normal in 11 (79%), borderline in 2 (14%), and delayed in 1 (7%), while PIQ was normal in 13 cases (93%) and borderline in one (7%). WMI and PSI evaluated in 11 children were impaired in 2 (18%, 1 borderline score and 1 delayed) and 7 (64%, 3 borderline score and 4 delayed) cases, respectively. See Table 2.

**Table 2:** Cognitive profile

**Sbj**	**Intelligence quotient**				
	**FSIQ**	**VIQ**	**PIQ**	**WMI**	**PSI**
1	93	96	102	***82***	97
2	94	104	100	100	***74***
3	93	96	102	-	-
4	86	112	***82***	100	***65***
5	***82***	***80***	98	***73***	***51***
6	***66***	***47***	91	-	-
7	***84***	***74***	100	91	88
8	94	108	93	97	***79***
9	112	130	95	124	***82***
10	94	126	100	91	***53***
11	106	118	108	115	***82***
12	121	126	130	109	88
13	91	108	111	88	107
14	95	100	90	-	-

Values are reported in standard scores (mean 100, SD 15) and categorized as follows: more than −1 DS normal, between − 1DS and −2 DS borderline, less than −2 DS delay. Bold-italic values represent borderline/delayed scores

*FSIQ* full-scale intelligence quotient, *PIQ* performance intelligence quotient, *PSI* processing speed index, *Sbj* subject, *VIQ* verbal intelligence quotient, *WMI* working memory index

Adaptive functioning was impaired in 3 children (17%), one of whom had difficulties in all the four VABS-II domains (communication, socialization, daily living skills, and motor skills), while the remaining 2 showed delay scores only in communication and daily living skills. See Table 3.

At CBCL composite scales, 5 (28%) children had risk score in total problems, 6 (33%) in internalizing problems, and 2 (11%) in externalizing problems. See Table 4. At CASD, 12 children (67%) showed one or more autistic-like features, even if none was in autism spectrum range (see Table 3).

**Table 3:** Adaptive functioning and behavioral aspects

**Sbj**	**Adaptive functioning (VABS-II), °p**					**Behavioral profile (CASD)**						
	**Total**	**COM**	**DLS**	**SOC**	**MS**	**SI**	**P**	**SS**	**C**	**M**	**AS**	**Total**
1	98	91	99	92	-	0/5	0/4	0/10	0/5	0/4	0/2	0/30
2	25	12	9	79	-	0/5	0/4	1/10	1/5	0/4	0/2	2/30
3	14	12	55	25	***3***	2/5	0/4	1/10	1/5	0/4	1/2	5/30
4	73	66	87	50	-	0/5	0/4	0/10	1/5	0/4	0/2	1/30
5	***3***	***1***	***3***	23	-	0/5	0/4	2/10	0/5	0/4	0/2	2/30
6	***0.2***	***0.5***	***2***	***3***	***0.1***	0/5	0/4	0/10	0/5	0/4	0/2	0/30
7	21	27	30	14	-	0/5	0/4	1/10	0/5	0/4	0/2	1/30
8	25	42	30	18	-	0/5	0/4	1/10	0/5	0/4	1/2	2/30
9	70	79	70	53	-	1/5	1/4	2/10	1/5	1/4	0/2	6/30
10	12	14	19	37	***5***	0/5	0/4	0/10	0/5	0/4	0/2	0/30
11	98	91	99	92	-	0/5	0/4	0/10	0/5	0/4	0/2	0/30
12	21	18	***5***	66	-	0/5	0/4	0/10	0/5	0/4	0/2	0/30
13	96	87	99	104	98	0/5	2/4	3/10	0/5	1/4	1/2	7/30
14	42	58	19	39	61	0/5	0/4	1/10	0/5	0/4	0/2	1/30
15	45	21	87	47	25	0/5	0/4	1/10	0/5	0/4	0/2	1/30
16	37	32	45	23	58	0/5	0/4	0/10	0/5	0/4	0/2	0/30
17	32	53	14	61	23	0/5	0/4	1/10	0/5	0/4	0/2	1/30
18	***5***	***3***	***5***	7	27	0/5	1/4	0/10	0/5	2/4	1/2	4/30

Bold-italic values represent deficient scores

*AS* problems with attention and safety, *C* atypical communication and development, *CASD* Checklist for Autism Spectrum Disorder, *COM* communication skills, *DLS* daily living skills, *M* mood, *MS* motor skills, *P* perseveration, *Sbj* subjects, *SI* problems with social interaction, *SOC* socialization, *SS* somatosensory disturbance, *VABS-II* Vineland Adaptive Behavior Scales-II. VABS scores: percentile (°p)

**Table 4:** Child behavior checklist (CBCL) results

	**Normal**	**Borderline**	**C. significant**
**CBCL 1½-5 and 6–18 composite scales** ** (** ***N*** ** = 18)**			
Total problems	13 (72)	3 (17)	2 (11)
Internalizing problems	12 (66)	3 (17)	3 (17)
Externalizing problems	16 (90)	1 (5)	1 (5)
**CBCL syndromes scales**			
CBCL 1½-5 (*N* = 5)			
Emotionally reactive	4 (80)	0	1 (20)
Anxious/depressed	4 (80)	1 (20)	0
Somatic complaint	5 (100)	0	0
Withdraw	5 (100)	0	0
Aggressive behavior	5 (100)	0	0
Attention problems	4 (80)	0	1 (20)
Sleep problems	5 (100)	0	0
CBCL 6–18 (*N* = 13)			
Anxious/depressed	10 (77)	3 (23)	0
Withdraw	10 (77)	2 (15)	1 (8)
Somatic complaint	12 (92)	0	1 (8)
Social problems	12 (92)	1 (8)	0
Though problems	13 (100)	0	0
Attention problems	11 (85)	2 (15)	0
Rule-breaking behavior	13 (100)	0	0
Aggressive behavior	13 (100)	0	0

*C. significant*, clinically significant. Values in brackets indicate percentages

### Correlations between visual acuity/genotype and phenotype (neurodevelopmental, cognitive, adaptive, and behavioral features)

Considering the cognitive level, we observed a statistically significant association between visual acuity and PIQ (*p*=0.001) and PSI (*p*=0.021). As regards adaptive functioning, statistical correlations were found between visual acuity and VABS-II total score (*p*=0.020), communication (*p*=0.020), and socialization (*p*=0.037) domains. No significant correlations were detected between visual acuity and emotional and behavioral difficulties at CBCL.

No significant correlations emerged between OCA1 and OCA2 subgroups according to the neurodevelopmental (motor milestones, *p*=0.06; language skills, *p*=0.1; and DQ at Griffiths-R, *p*=0.1), cognitive (FSIQ, *p*=0.5), adaptive (total score at VABS-II, *p*=0.9), and behavioral (total score at CASD, *p*=0.5; CBCL total problems, *p*=0.6; internalizing *p*=0.3; and externalizing *p*=0.5) profiles.

## Discussion

OCA is a rare genetic disorder caused by the complete absence or reduction of biosynthesis of melanin in melanocytes resulting in mild to severe depigmentation of the hair, skin, and eyes [3]. Therefore, it is not surprising that research on subjects affected by OCA has been mainly focused on cutaneous and ocular signs and symptoms. To date, the neurodevelopmental profile has been poorly investigated, although several evidences support the hypothesis that the brain may be involved in OCA: directly, due to the disruption of the mechanisms involved in the conversion of tyrosine in molecules essential for the retinal and visual network development [40], and indirectly, as a consequence of congenital and often severe visual problems that could interfere with neurodevelopment, as documented in OA children [22] and in other clinical populations [24, 41]. In the present study, we aimed at detailing the neurological and neurovisual skills, cognitive level, adaptive functioning, and behavioral aspects of children with OCA, also exploring a possible correlation between the level of visual acuity deficit/genotype (OCA1 vs OCA2) and the clinical profile.

About half of the children (56%) presented an impairment in global neurodevelopment at an early stage. This percentage seems to be higher than that reported in pediatric population-based studies (neurodevelopmental delay between 4.5 and 12%) [42], and similar to that observed in children with Leber’s congenital amaurosis (45%), “other” congenital retinal dystrophies (33%), or congenital disorders of the peripheral visual system (38–61%) [43, 44]. However, these studies used different measures to evaluate the neurodevelopment, so it is difficult to compare the data. All the developmental areas of our children were affected, specifically gross motor, personal–social, communicative, hand–eye coordination, and performance domains. It has been hypothesized that an early and severe visual deficit may affect the acquisition of motor skills [45, 46], daily life and social abilities [47], language [48], and cognition [43]. Children typically develop through the interaction with the environment, a process mainly mediated by vision. In the absence of the “incentive” represented by the sight [49], children may have difficulties to build up a picture of the world [45] and develop gross and fine motor skills, above all during the second 6 months of life, when the movements begin to be enhanced by voluntary patterns and relational aspects (exploration, inquiry, etc.). In this regard, some of our children presented a slight delay in achieving head control and sitting position, and a marked delay for the other developmental milestones, such as walking. The development of prehension may be also impaired since the hands of the visually impaired child remain at shoulder height for a longer time [45] without the natural behavior of environmental exploration. Furthermore, hand–eye coordination could be achieved later due to its dependence on hearing cue [50, 51]. Our children appropriately acquired the early fine motor milestones, later showing difficulties in hand–eye coordination, probably because the visual acuity deficit was not so severe as to limit the explorative tactile function of their hands. The peculiar perceptual experience due to visual deficits may also limit the language development [48], with difficulties in the construction of lexical semantics, which implies the construction of a relationship between language and extra-linguistic reality. The attribution of lexical labels to familiar people and objects needs the possibility of the child to look at what adult is looking at by following the direction of his gaze and thus associate signifier with signified. Therefore, the impairment in referential looking slows down lexical acquisition [52, 53]. In line with literature, 38% of our children presented a delay in word production and/or vocabulary expansion evolving in two cases in receptive and expressive language disorder. By contrast, in the general population, approximately 15% of children have slow onset and progression of expressive language [54, 55], and 5–7% present a developmental language disorder [56, 57]. Vision also exerts a crucial role in the socio-relational skill development: eye contact represents the earliest mediator of mother–infant communication [58], and thus, a visual impairment may lead to a distortion of this relationship resulting in personal–social difficulties as observed in our sample and in literature [58]. Finally, vision is considered the most important sense for the construction of sensorimotor intelligence [59]: through vision, the child acquires the most important cognitive milestones such as the awareness of physical causality, spatial relations, and object permanence. An early-onset visual deficit may determine difficulties in the cognitive processing of reality. These data may explain why our children showed delayed score in performance and global domains at GMDS-R.

At the time of direct evaluation, all the children presented a normal neurological examination except for receptive and expressive language disorder (2 cases) and for a variable combination of visual signs and symptoms, in particular refractive errors, iris translucency, hypopigmentation of the retina, foveal hypoplasia, photophobia, abnormal head position, strabismus, nystagmus, unstable fixation, discontinued smooth pursuit, saccades dysfunctions, reduced visual acuity (moderate visual impairment/partial blindness), altered contrast sensitivity, and visual field limitations. Literature data report that these ocular, oculomotor, and basic visual functions manifestations are shared across all types of OCA [60].

We observed that the delayed score in DQ at GMDS-R did not evolve into an intellectual disability. The only child with delayed scores at FSIQ presented a PIQ within normal range and a severe language disorder that can justify the low score at verbal and total scale. Literature data on cognitive skills in subjects suffered from peripheral visual deficit are dated and scarce, reporting different results according to etiopathogenesis: studies conducted on albino subjects did not detect intellectual deficits [16–18] and, thus, support our observations, while cognitive disability was found in patients with Leber’s congenital amaurosis (50%), probably for an association with central nervous system (CNS) anomalies (such as microgyria, polygyria, hypoplasia of the cerebellar vermis, ventricular dilatation) [61]. In the presence of a visual impairment, sound and touch can guide motor experience and exploration of the surrounding environment. The “reach and touch on sound” stage, i.e., the ability to reach out for and grasp an object presented exclusively through the sound, constitutes a topical moment in the development of visually impaired children, allowing them to access to the world of mental representation [45, 49]. Literature indicates that by the age of 36 months, most children with visual impairment fully acquire the reach on sound function, considered a condition of, and a catalyst to, all the subsequent achievements [45]. In this regard, we hypothesize that the achievement of the reach on sound phase in our sample may have organized not only motor, but also cognitive and behavioral experience, determining a normal developmental trajectory. Furthermore, our data are in line with literature, reporting normal cognitive quotients in albino subjects [16–18]. The level of visual acuity seemed to positively correlate with PIQ and PSI, probably because these scales may be more dependent from visual input.

Few subjects of our sample reported difficulties in adaptive skills according to the VABS-II interview. No data on adaptive functioning of individuals affected by OCA are available, but studies on visually impaired subjects documented inadequate skills in the majority of cases [62, 63]. This difference may be explained by the characteristics of sample in terms of (1) the level of visual acuity (predominantly blindness in the above cited studies vs predominantly low vision in ours), (2) timing of visual deficit onset (different age vs congenital), and (3) etiology (multiple causes vs OCA). As described by other authors [64–66], we found that the level of visual acuity seemed to influence the VABS-II total score, as well as the communicative and social functioning domains: subjects with marked visual impairment had greater adaptive difficulties. Different factors may contribute to this positive relation. First, children with a basic level of vision may have a protection to social communicative development compared to those with profound visual impairment [65]. In fact, vision could be considered as a mediator of verbal language development (as described above) as well as of non-verbal pragmatic behaviors of social interactions (such as eye contact, recognition of facial expression, and visual imitation) [65, 67]. Second, out-of-school activities such as socializing, dancing, playing non-team sports, and going to movies, depends on sensory input, and thus, impairments may lead to lower performance in daily life skills and socialization [47, 62]. Furthermore, parents tend to be overprotective with their child, especially if blind, limiting them in coping with tasks by themselves [62, 64].

According to the CBCL, 33% of our children reached the clinical or subclinical range for internalizing problems, percentage that is higher than that observed in healthy pediatric population (anxiety disorders: 6.5%; depressive disorder: 2.6%) [68]. Our findings mirror previous studies that reported higher risk of emotionally reactive, anxiety, avoidant, and withdrawal behaviors in peripheral visually impaired children (internalizing problems: 22.7%) [69], although the exact mechanisms of this heightened risk are still unclear. It has been suggested that a visual reduction and the resulting limited sensory experiences may have a negative impact on emotional–behavioral self-regulation [70, 71]. More than half of our children (67%) showed one or more autistic-like features. This prevalence is similar to that reported in children with optic nerve hypoplasia and septo-optic dysplasia (58%) [72] and higher compared to congenital disorders of the peripheral visual system (20%) [38] and pediatric population (12.5%) [73]. However, none of our patients reached the cut-off criteria at CASD questionnaire for an ASD. The presence of a diagnosis of ASD in visually impaired children greatly varies among studies and has been reported in 31% of subjects with optic nerve hypoplasia and septo-optic dysplasia [72], 8–12% of children with congenital disorders of the peripheral visual system [38, 74], and in 1% of general population [75]. These differences may be explained by methodological heterogeneity (different instruments for evaluating autistic-related behaviors) and by the characteristics of samples (all children with albinism in our cohort and mixed form of peripheral visual impairment in the other studies). Given the occurrence of autistic-like features in visually impaired children, we hypothesize that these traits may reflect a blind-specific developmental problem in the acquisition of socio-cognitive abilities rather than a specific ASD [22, 38, 76].

Finally, we did not find a specific neurodevelopmental, cognitive, adaptive, and behavioral profile according to the genetic defect (OCA1 vs OCA2). The absence of significant genotype–phenotype correlations has been also reported in a recent study conducted by Dumitrescu and colleagues (2021) on visual features in a cohort of patients with OCA [77].

In conclusion, our findings suggest that children with OCA may present an early neurodevelopmental delay that tend to be solved with age. The adaptive functioning seemed to be spared, but the level of visual acuity may influence the communicative and social abilities as well as the global adaptive behavior. Subjects may show risks for emotional–behavioral problems, as well as autistic-like features that need to be correctly judged to avoid an improper diagnosis [38, 76]. We are aware that it is difficult to compare data due to differences in methodologies among studies, but the neurocognitive difficulties found in our sample are similar to those of children with peripheral visual impairment. Furthermore, it has been demonstrated the presence of CNS anomalies in albino subjects, such as an increase in cortical thickness of the occipital cortex similar to that found in peripherally blind subjects, suggesting an association between degraded visual input and changes in brain morphology [78]. Therefore, we hypothesize that the brain is particularly vulnerable to the effects of visual impairment. Nonetheless, alterations of the genes involved in albinism may play a role in the neurodevelopmental problems, since it has been documented their expression also in CNS [79, 80], but their role has not yet been clearly understood. Although it has been described that the association between OCA and neurodevelopmental delay might indicate the presence of Prader–Willi (PWS) or Angelman syndrome (AS) [81], due to the adjacency of *OCA2* and *UBE3A* gene on chromosome 15, we exclude that neurocognitive profile observed in our children may be related to these conditions, because we found (1) uneventful perinatal period (feeding problems and failure to thrive are reported in PWS); (2) mild motor, language, and neurodevelopmental delay (“severe” in AS); (3) absence of intellectual disability (one of the hallmarks of AS and PWS); and (4) no dysmorphic features and/or neurological signs and symptoms (such as hypotonia reported in PWS or microcephaly and movement or balance disorders in AS) [82, 83]. The neurocognitive signs observed in our cohort underline the importance of an early neuropsychiatric evaluation to identify those children with neurodevelopmental/psychological difficulties and to adopt habilitative interventions.

The small sample size and the heterogeneity regarding age are the two main limitations of the present study that do not allow the generalization of the findings. Although OCA is a rare genetic disease, future studies should be conducted in a larger cohort of participants to better characterize these aspects.

## Supplementary Information

Below is the link to the electronic supplementary material.

Supplementary file1 (DOC 70 KB)

## Data Availability

The raw data supporting the conclusions of this article will be made available by the authors, without undue reservation.

## Abbreviations

ASD: Autism spectrum disorders
CASD: Checklist for Autism Spectrum Disorder
CBCL: Child Behavior Checklist
CNS: Central nervous system
DQ: Developmental quotient
FSIQ: Full-scale intelligence quotient
GMDS-R: Griffiths Mental Developmental Scales–Revised
OA: Ocular albinism
OCA: Oculocutaneous albinism
PRI: Perceptual reasoning index
PIQ: Performance intelligence quotient
PSI: Processing speed index
VCI: Verbal comprehension index
VIQ: Verbal intelligence quotient
VABS: Vineland Adaptive Behavior Scales
WPPSI-III: Wechsler Preschool and Primary Scale of Intelligence III edition
WISC-IV: Wechsler Scales of Intelligence for Children IV edition
WMI: Working memory index
WHO: World Health Organization
