# Supplementary material for: Oculocutaneous albinism: the neurological, behavioral, and neuro-ophthalmological perspective
Source: Eur J Pediatr. 2023 Apr 3;182(6):2723–33. doi: 10.1007/s00431-023-04938-w (PMC10257606; doi:10.1007/s00431-023-04938-w)
Supplement: Supplementary file 1 — Supplementary file1 (DOC 70 KB) [file 431_2023_4938_MOESM1_ESM.doc]

**Oculocutaneous albinism: the neurological, behavioral, and neuro-ophthalmological perspective**

Jessica Galli^1,2^*, Erika Loi^2,3^, Laura Dusi^1^, Nadia Pasini^4^, Andrea Rossi^2^, Vera Scaglioni^2^, Lucia Mauri^5^, Elisa Fazzi^1,2^

^1^Department of Clinical and Experimental Sciences, University of Brescia, Brescia, Italy

^2^Unit of Child Neurology and Psychiatry, ASST Spedali Civili of Brescia, Brescia, Italy

^3^Department of Molecular and Translational Medicine, University of Brescia, Brescia, Italy

^4^Department of Neurological and Vision Sciences, ASST Spedali Civili of Brescia

^5^Medical Genetics Unit, Department of Laboratory Medicine, ASST Grande Ospedale Metropolitano Niguarda, Milan, Italy.

*Correspondence

Corresponding Author: Jessica Galli; E-mail: jessica.galli@unibs.it

**Abstract**

Purpose: Oculocutaneous albinism (OCA) is a group of rare, genetic disorders caused by absent/reduced melanin biosynthesis. The aim of this study was to explore the neurovisual, cognitive, adaptive, and behavioral profile of children affected by OCA, also evaluating any possible effect of the visual acuity deficit on the clinical profile and genotype-phenotype correlations.

Methods: Eighteen children (9 males, mean age 84~~.5~~ months ±41~~.2~~; range 18-181 months) with a molecular confirmed diagnosis of OCA were enrolled in the study. We collected data on clinical history, neurodevelopmental profile, neurological and neurovisual examination, cognitive, adaptive and emotional/behavioral functioning.

Results: A global neurodevelopmental impairment was detected in 56% of the children, without evolving into an intellectual disability. All the patients showed signs and symptoms of visual impairment. Low adaptive functioning was observed in 3 cases (17%). A risk for internalizing behavioral problems was documented in 6 cases (33%), for externalizing problems in 2 (11%) and for both in 5 (28%). Twelve children (67%) showed one or more autism-like features. Correlation analyses revealed significant associations between the visual acuity level and Performance Intelligence Quotient (*p*=0.001), Processing Speed Index (*p*=0.021), Vineland total score (*p*=0.020), Vineland communication (*p*=0.020) and socialization (*p*=0.037) domains. No significant correlations were found between genotype and phenotype.

Conclusions: Children with OCA may present a global neurodevelopmental delay that seems to improve with age, and emotional/behavioral difficulties, along with the well-known visual impairment. An early neuropsychiatric evaluation and habilitative training are recommended to improve vision-related performance, neurodevelopment, and any psychological difficulties.

Keywords: albinism; children; neurodevelopment; vision; intelligence quotient.

**What is known**

- Children with oculocutaneous albinism show dermatological and ophthalmological problems.
- An early visual impairment may have negative implications on motor, emotional and mental processes that would allow the child to organize his or her experiences.

**What is new**

- In addition to a variable combination of ocular signs and symptoms, children with oculocutaneous albinism may present an early neurodevelopmental delay and emotional/behavioral difficulties.
- An early visual treatment are recommended to improve vision-related performance, neurodevelopment, and any psychological difficulties.

**List of Abbreviations in alphabetical order**

ASD: Autism spectrum disorders

CASD: Checklist for Autism Spectrum Disorder

CBCL: Child Behavior Checklist

CNS: Central nervous system

DQ: Developmental Quotient

FSIQ: Full Scale Intelligence Quotient

GMDS-R: Griffiths Mental Developmental Scales–Revised

OA: Ocular albinism

OCA: Oculocutaneous albinism

PRI: Perceptual Reasoning Index

PIQ: Performance Intelligence Quotient

PSI: Processing Speed Index

VCI: Verbal Comprehension Index

VIQ: Verbal Intelligence Quotient

VABS: Vineland Adaptive Behavior Scales

WPPSI-III: Wechsler Preschool and Primary Scale of Intelligence III edition

WISC-IV: Wechsler Scales of Intelligence for Children IV edition

WMI: Working Memory Index

WHO: World Health Organization

**INTRODUCTION**

Oculocutaneous albinism (OCA) is a rare, autosomal recessive disorder caused by the complete absence or reduction of biosynthesis of melanin in melanocytes. It affects people globally, with an overall prevalence of approximately 1 in 20.000 [1], with different rates across geographic regions and ethnic groups [2].

Individuals affected by OCA present a normal number of melanocytes in the epidermis and follicles, but they totally or partially lack of the melanin pigment [3]. Currently, eight forms have been identified (from OCA1 to OCA8) with a highly variable phenotype [1; 4-5]. Ocular abnormalities, such as hypopigmentation of iris and retina, foveal hypoplasia, and atypical decussation at the optic chiasm, also characterize the disorder, resulting in a wide spectrum of visual signs and symptoms, such as reduced visual acuity, impaired stereopsis, strabismus, nystagmus, iris translucency, and photophobia [6].

Several alterations in genes encoding proteins involved in the melanin biosynthesis pathway have been identified: specifically, the *TYR* gene (OCA1, both A and B type), *OCA2* or *P* gene (OCA2), *TYRP1* (OCA3) [7], *SLC45A2* (OCA4) [4], *SLC24A5* (OCA6) [8], *LRMDA* (OCA7) [9] and *~~TYRP2~~* DCT (OCA8) [~~2~~ 10]; the genetic mutation for the OCA5 subtype has not yet been identified, but mapped to chromosome 4q24 [~~10~~ 11]. Finally, OCA can also occur in several syndromic disorders, such as the Hermansky-Pudlak syndrome [~~11~~ 12] and the Chediak-Higashi syndrome [~~12~~ 13].

To date, neurodevelopmental outcomes in subjects with OCA are poorly investigated and the literature is limited to few studies [~~13-17~~ 14-18] and case reports [~~18-20~~ 19-21]. Kutzbach and colleagues [~~13-~~14-15] reported the presence of attention-deficit/hyperactivity disorder in 6.8% of adults and in 21.7% of children with OCA, and of autism spectrum disorders (ASDs) in 3.8% of children; no reading disorders were found in the 44 children evaluated. As regard cognitive profile, normal quotient has been reported in albino subjects [~~15-17~~ 16-18]. Finally, in a recent study by our group on children with ocular albinism (OA) [~~21~~ 22], we documented neurodevelopmental problems (such as developmental delay, impaired cognitive profile, language disorder, and autistic-like features) above all in children with a genetically confirmed OA diagnosis. The association between OCA and neurodevelopmental disorders could be explained by genetic factors (mutation of the *GABRA5* gene, candidate for autism and located near to the *OCA2* gene) [~~19~~ 20] and brain connectivity dysfunctions (misdirection in central nervous system networks other than the visual pathways) [~~13; 20-21~~ 14; 21-22].

Despite some descriptive data documenting the cognitive level and academic skills of albino subjects, no studies, to our knowledge, provide a comprehensive description of their clinical profile, collecting and relating different developmental areas such as neurovisual, cognitive, adaptive and behavioural features. Given these premises, the aim of the present study was 1) to explore these neurodevelopmental areas that characterized children affected by OCA, 2) to evaluate any possible effect of the visual acuity deficit on cognitive, adaptive and behavioural functions, and 3) to explore any possible genotype-phenotype correlations.

**METHODS**

We collected and analyzed demographic, genetical, and clinical data on 18 children (9 males, mean age of 84~~.5~~ months, SD 41~~.2~~; range 18-181 months), referred to our Neuro-ophthalmological Tertiary Centre, Child Neurology and Psychiatry Unit, ASST Spedali Civili of Brescia between January 2021 and July 2022. Inclusion criteria were the diagnosis of OCA, confirmed by genetic analysis, and age from birth to 18 years. Exclusion criteria were the presence of syndromic forms of albinism or ocular albinism. ~~All subjects were eligible for the present study and none declined to participate.~~ None of the eligible subjects declined to participate.

Data on clinical history (pregnancy, delivery, birth weight, and neurodevelopment milestones) and on neurodevelopmental profile, evaluated using the Griffiths Mental Developmental Scales–Revised (GMDS-R) [~~22~~ 23], were collected from medical charts. Furthermore, the enrolled children underwent neurological and neurovisual examination, cognitive evaluation, adaptive and emotional/behavioral functioning assessments.

The neurovisual profile was assessed according to our protocol [~~23-25~~ 24-26] that includes the evaluation of: ophthalmological characteristics (refraction under cycloplegia, anterior segment and ocular fundus); oculomotor functions as fixation (defined as altered when unstable or absent), smooth pursuit (“altered” when discontinuous or difficult to elicit/absent), saccadic movements (“altered” when dysmetric and/or with increased latency or absent), strabismus, ocular motility deficit and abnormal eye movements; basic visual functions (visual acuity, contrast sensitivity, visual field). Visual acuity was evaluated under maximum refractive correction with test suitable for patient’s age and cooperation using Teller Acuity Cards [~~26~~ 27], Lea Symbols or letter optotypes [~~27~~ 28]. We defined visual acuity score as normal or reduced according to normative data [~~26; 28-29~~ 27; 29-30]. Contrast sensitivity was evaluated using the Hiding Heidi Low Contrast “Face” Test and considered as “reduced” if > 2.5%, while the visual field was evaluated through the kinetic perimetry [~~30~~ 31].

Cognitive level was evaluated using age-appropriate versions of Wechsler Preschool and Primary Scale of Intelligence III edition (WPPSI-III) [~~31~~ 32] and Wechsler Scales of Intelligence for Children IV edition (WISC-IV) [~~32~~ 33]. Full Scale Intelligence Quotient (FSIQ), Verbal Comprehension/Verbal Intelligence Quotient (VCI/VIQ), Perceptual Reasoning/Performance Intelligence Quotient (PRI/PIQ), Working Memory (WMI), and Processing Speed Index (PSI) scores were collected. All the quotients are reported in standard scores (mean 100, SD 15) and defined as follow: more than −1 DS, normal; between −1 and −2 DS, borderline; <-2 DS, delay.

Adaptive functioning was evaluated using the Vineland Adaptive Behavior Scales-II (VABS-II) [~~33~~ 34], a questionnaire filled out by parents that covers four domains of adaptive behavior, that are: communication, socialization, daily living skills, and motor skills. Centile score <5 was considered as a cut off for adaptive disorders.

Emotional and behavioral characteristics were assessed using the parent report Child Behavior Checklist (CBCL) 1½-5 [~~34-~~35-36] or 6–18 [~~36~~ 37] according to age. Composite scales (Internalizing, Externalizing, and Total problems) and syndrome scales (CBCL 1½-5: Emotionally reactive, Anxious/Depressed, Somatic complaints, Withdrawn, Sleep problems, Attention problems, and Aggressive behavior; CBCL 6–18: anxious/depressed, withdrawn/ depressed, somatic complaints, social problems, thought problems, attention problems, rule-breaking behavior and aggressive behavior) are obtained from the item scores, categorized as “normal,” “borderline,” or “clinically significant” according to the tool kit software standards. Given the high frequency of autism-like features in visually impaired children [~~37~~ 38] and in children with ocular albinism [~~21~~ 22], we performed the Checklist for Autism Spectrum Disorder (CASD) [~~38~~ 39]. Scores of 15 or higher are within the autism range, scores of 10-14 in the autism spectrum range, and scores less than 10 characterize children with typical development or different diagnoses.

The study was conducted in accordance with the ethical guidelines set forth by the Declaration of Helsinki and was approved by the Ethical Committee of ASST Spedali Civili in Brescia, Italy (NP 5523). Written informed consent was obtained from parents/caregivers of the children.

**STATISTICAL ANALYSIS**

A descriptive analysis of the findings was performed. Qualitative variables were analyzed in terms of number and percentage, while quantitative data were reported as mean, standard deviation and range. The Pearson or Spearman correlation analysis has been performed using SPSS software to evaluate the impact of the visual acuity deficit on cognitive level, adaptive functioning, and emotional/behavioral profile. Given limitations in genotype incidence, we were able to compare only two genotypes: OCA1 and OCA2. We applied a Fisher’s Exact Test to study the relationship between neurodevelopmental, cognitive, adaptive, and behavioral aspects and genetic defects. A p-value below 0.05 was interpreted as statistically significant. When missing data were present due to the age of the children, we excluded from the analyses of the specific variable those subjects with missing data.

**RESULTS**

*3.1 Data collection*

Out of 18 subjects enrolled, 10 children (56%) had a *TYR* gene alteration (OCA1), 7 (38%) an *OCA2* gene deletion or mutation (OCA2) and 1 (6%) a *SLC45A2* gene deletion (OCA4).

From the medical charts revision, pregnancy was uneventful in 16 (89%) cases while the mothers of 2 children (11%) suffered from gestational diabetes. Delivery was at term in 17 (94%) cases while a boy was born at 35 weeks of gestation. The mean birth weight was 3129.5 ± 426.0 grams (range: 2540-4000 grams). Perinatal was uneventful in all the sample.

Eight (44%) children appropriately acquired the gross (head control, sitting, walking) motor milestones, while 10 (56%) had different levels of delay: 5 (28%) presented with a delay in the head control, 5 (28%) in the sitting position, 4 (22%) in the walking, and 2 (11%) an overall delay. Specifically, the mean age of head control was 3~~.2~~ ± 1~~.2~~ months (range 2-6 months), sitting 7~~.5~~ ± 2~~.3~~ (range 5-12 months), walking ~~15.9~~ 16 ± 4~~.2~~ (range: 10-24 months). Thumb finger was appropriately acquired in all the cases (mean age 12~~.5~~ ± ~~0.7~~ 1 months, range 12-14 months). Data on communication and language skills were collected in 16 children; the remaining two were infants (aged between 18-21 months) and have not yet achieved the vocabulary expansion. Six (38%) children were *Late Talkers* because they produced fewer than 50 words or no word combinations at 24 months of age. Four of these eventually met their same-age peers in language performance and therefore they can be considered *Late Bloomers*; the remaining two *Late Talkers* received a diagnosis of Language Disorder at age six because they showed impairments in both receptive and expressive language skills on specific neuropsychological test. More in general, canonical babbling was acquired at 8~~.2~~ ± 1~~.4~~ months (range 7-12 months), 1^st^ word at 16~~.2~~ ± 5~~.4~~ (range 10-27 months), and >50 words at 30~~.1~~ ± ~~9.8~~ 10 months (range 24-60 months).

The Developmental profile assessment evaluated in 16 children using GMDS-R (mean age at evaluation: ~~18.8~~ 19 months ± 12~~.3~~, range 6-24) revealed normal scores on Developmental Quotient (DQ) in 7 cases (44%), borderline score in 5 (31%) and delayed score in 4 (25%). The remaining two children referred to the Center in older ages and, therefore, did not undergo the GMDS-R assessment. The analysis of each domain revealed borderline/delay scores as follow: Locomotor (3 borderline, 4 delay), Personal-Social (2 borderline, 6 delay), Hearing-Speech (2 borderline, 5 delay), Eye-Hand Coordination (2 borderline, 7 delay), Performance (3 borderline, 5 delay).

*3.2 Direct assessment*

Direct assessment was carried out at mean age of 84~~.5~~ months (SD 41~~.2~~; range 18-181 months). No abnormalities concerning cranial nerve, head circumference, muscle strength, tone and bulk, reflexes and gait were detected.

From an ophthalmological perspective, all the 18 (100%) children presented refractive errors. Iris translucency and ocular fundus abnormalities characterized by hypopigmentation of retina and/or foveal hypoplasia were detected in 17 (94%) and 18 (100%) children, respectively. Eight children (44%) had photophobia and 16 (89%) an abnormal head position. As regards oculomotor functions, strabismus was observed in 14 (78%), nystagmus in 18 (100%), and alterations in fixation, smooth pursuit, and saccadic movements in 18 (100%), 17 (94%) and 13 (72%) children, respectively. The visual acuity was reduced in all the sample (100%), contrast sensitivity in 6 (33%) and visual field in 6 (33%). See Table 1.

**Table 1 - Neurovisual findings of the sample.**

| **Sbj** | **Refr.**  **Errors** | **Ny** | **Strabismus** | **Fixation** | **Smooth**  **pursuit** | **Saccades** | **Visual acuity** | **Contr**  **Sens.** | **Visual**  **Field** |
| --- | --- | --- | --- | --- | --- | --- | --- | --- | --- |
| 1 | As, H | Pend | Exo | A | A | A | 0.2 | 1.25 | N |
| 2 | As, M | Jerky | No | A | A | A | 0.06 | 2.5 | N |
| 3 | As, M | Pend | Eso | A | A | A | 0.05 | 1.25 | N |
| 4 | As, M | Pend | Eso | A | A | A | 0.1 | 1.25 | A |
| 5 | As, H | Pend | Exo | A | A | N | 0.1 | 1.25 | A |
| 6 | As, M | Pend | Eso | A | A | A | 0.1 | 5 | A |
| 7 | As, H | Pend | Exo | A | A | A | 0.15 | 1.25 | N |
| 8 | As, H | Pend | Eso | A | A | A | 0.1 | 1.25 | A |
| 9 | As, M | Mix | Eso | A | A | A | 0.1 | 1.25 | N |
| 10 | As, M | Jerky | Exo | A | A | A | 0.05 | 1.25 | N |
| 11 | As, M | Mix | Exo | A | A | N | 0.15 | 1.25 | A |
| 12 | As, H | Pend | Exo | A | A | A | 0.3 | 1.25 | N |
| 13 | As, H | Jerky | Eso | A | N | A | 0.2 | 1.25 | N |
| 14 | As | Pend | No | A | A | N | 3.1* | 1.25 | N |
| 15 | As | Mix | No | A | A | A | 4.7* | 5 | N |
| 16 | As, M | Mix | No | A | A | A | 4.7* | 5 | N |
| 17 | As, M | Pend | Eso | A | A | N | 3.1* | 5 | A |
| 18 | As, M | Pend | No | A | A | N | 0.9* | 100 | N |

Legend: A, altered; As, astigmatism; Contr. Sens., contrast sensitivity; Eso, esotropia; Exo, exotropia; H, hypermetropia; M, myopia; N, normal; Ny, nystagmus; Pend, pendular; Refr. Errors, refractive errors.

*values are reported in cyc/deg.

The cognitive evaluation was performed in 14 children (the remaining four subjects were too young for the WPPSI/WISC-IV assessment) and showed normal FSIQ in 11 children (79%), borderline score in 2 cases (14%) and deficient in 1 (7%). VIQ was normal in 11 (79%), borderline in 2 (14%) and delayed in 1 (7%) while PIQ was normal in 13 cases (93%) and borderline in one (7%). WMI and PSI evaluated in 11 children were impaired in 2 (18%, 1 borderline score and 1 delayed) and 7 (64%, 3 borderline score and 4 delayed) cases respectively. See Table 2.

**Table 2 - Cognitive profile.**

| **Sbj** | **Intelligence quotient** | | | | |
| --- | --- | --- | --- | --- | --- |
|  | **FSIQ** | **VIQ** | **PIQ** | **WMI** | **PSI** |
| 1 | 93 | 96 | 102 | ***82*** | 97 |
| 2 | 94 | 104 | 100 | 100 | ***74*** |
| 3 | 93 | 96 | 102 | - | - |
| 4 | 86 | 112 | ***82*** | 100 | ***65*** |
| 5 | ***82*** | ***80*** | 98 | ***73*** | ***51*** |
| 6 | ***66*** | ***47*** | 91 | - | - |
| 7 | ***84*** | ***74*** | 100 | 91 | 88 |
| 8 | 94 | 108 | 93 | 97 | ***79*** |
| 9 | 112 | 130 | 95 | 124 | ***82*** |
| 10 | 94 | 126 | 100 | 91 | ***53*** |
| 11 | 106 | 118 | 108 | 115 | ***82*** |
| 12 | 121 | 126 | 130 | 109 | 88 |
| 13 | 91 | 108 | 111 | 88 | 107 |
| 14 | 95 | 100 | 90 | - | - |

Legend: FSIQ, full-scale intelligence quotient; PIQ, performance intelligence quotient; PSI, processing speed index; Sbj, subject; VIQ, verbal intelligence quotient; WMI, working memory index.

Values are reported in standard scores (mean 100, SD 15) and categorized as follow: more than -1DS, normal; between -1DS and -2DS, borderline; less than -2DS, delay.

Adaptive functioning was impaired in 3 children (17%), one of whom had difficulties in all the four VABS-II domains (communication, socialization, daily living skills and motor skills) while the remaining 2 showed delay scores only in communication and daily living skills. See Table 3.

At CBCL composite scales, 5 (28%) children had risk score in total problems, 6 (33%) in internalizing problems and 2 (11%) in externalizing problems. See Table 4. At CASD, 12 children (67%) showed one or more autistic-like features, even if none were in autism spectrum range (see Table 3).

**Table 3 - Adaptive functioning and behavioral aspects.**

| **Sbj** | **Adaptive functioning (VABS II), °p** | | | | | **Behavioral profile (CASD)** | | | | | | |
| --- | --- | --- | --- | --- | --- | --- | --- | --- | --- | --- | --- | --- |
|  | **Total** | **COM** | **DLS** | **SOC** | **MS** | **SI** | **P** | **SS** | **C** | **M** | **AS** | **Total** |
| 1 | 98 | 91 | 99 | 92 | - | 0/5 | 0/4 | 0/10 | 0/5 | 0/4 | 0/2 | 0/30 |
| 2 | 25 | 12 | 9 | 79 | - | 0/5 | 0/4 | 1/10 | 1/5 | 0/4 | 0/2 | 2/30 |
| 3 | 14 | 12 | 55 | 25 | 3 | 2/5 | 0/4 | 1/10 | 1/5 | 0/4 | 1/2 | 5/30 |
| 4 | 73 | 66 | 87 | 50 | - | 0/5 | 0/4 | 0/10 | 1/5 | 0/4 | 0/2 | 1/30 |
| 5 | 3 | 1 | 3 | 23 | - | 0/5 | 0/4 | 2/10 | 0/5 | 0/4 | 0/2 | 2/30 |
| 6 | 0.2 | 0.5 | 2 | 3 | 0.1 | 0/5 | 0/4 | 0/10 | 0/5 | 0/4 | 0/2 | 0/30 |
| 7 | 21 | 27 | 30 | 14 | - | 0/5 | 0/4 | 1/10 | 0/5 | 0/4 | 0/2 | 1/30 |
| 8 | 25 | 42 | 30 | 18 | - | 0/5 | 0/4 | 1/10 | 0/5 | 0/4 | 1/2 | 2/30 |
| 9 | 70 | 79 | 70 | 53 | - | 1/5 | 1/4 | 2/10 | 1/5 | 1/4 | 0/2 | 6/30 |
| 10 | 12 | 14 | 19 | 37 | 5 | 0/5 | 0/4 | 0/10 | 0/5 | 0/4 | 0/2 | 0/30 |
| 11 | 98 | 91 | 99 | 92 | - | 0/5 | 0/4 | 0/10 | 0/5 | 0/4 | 0/2 | 0/30 |
| 12 | 21 | 18 | 5 | 66 | - | 0/5 | 0/4 | 0/10 | 0/5 | 0/4 | 0/2 | 0/30 |
| 13 | 96 | 87 | 99 | 104 | 98 | 0/5 | 2/4 | 3/10 | 0/5 | 1/4 | 1/2 | 7/30 |
| 14 | 42 | 58 | 19 | 39 | 61 | 0/5 | 0/4 | 1/10 | 0/5 | 0/4 | 0/2 | 1/30 |
| 15 | 45 | 21 | 87 | 47 | 25 | 0/5 | 0/4 | 1/10 | 0/5 | 0/4 | 0/2 | 1/30 |
| 16 | 37 | 32 | 45 | 23 | 58 | 0/5 | 0/4 | 0/10 | 0/5 | 0/4 | 0/2 | 0/30 |
| 17 | 32 | 53 | 14 | 61 | 23 | 0/5 | 0/4 | 1/10 | 0/5 | 0/4 | 0/2 | 1/30 |
| 18 | 5 | 3 | 5 | 7 | 27 | 0/5 | 1/4 | 0/10 | 0/5 | 2/4 | 1/2 | 4/30 |

Legend: AS, problems with attention and safety; C, atypical communication, and development; CASD, checklist for autism spectrum disorder; COM, communication skills; DLS, daily living skills; M, mood; MS, motor skills; P, perseveration; Sbj, subjects; SI, problems with social interaction; SOC, socialization; SS, somatosensory disturbance; VABS II, Vineland Adaptive Behavior Scales II.

VABS scores: percentile (°p).

**Table 4 - Child behavior checklist (CBCL) results.**

|  | **Normal** | **Borderline** | **C. significant** |
| --- | --- | --- | --- |
| **CBCL 1½-5 and 6-18 composite scales** (*N*=18)  Total problems  Internalizing problems  Externalizing problems | 13 (72)  12 (66)  16 (90) | 3 (17)  3 (17)  1 (5) | 2 (11)  3 (17)  1 (5) |
| **CBCL syndromes scales**  CBCL 1½-5 (*N*=5)  Emotionally reactive  Anxious/depressed  Somatic complaint  Withdraw  Aggressive behavior  Attention problems  Sleep problems  CBCL 6-18 (*N*=13)  Anxious/depressed  Withdraw  Somatic complaint  Social problems  Though problems  Attention problems  Rule-breaking behavior  Aggressive behavior | 4 (80)  4 (80)  5 (100)  5 (100)  5 (100)  4 (80)  5 (100)  10 (77)  10 (77)  12 (92)  12 (92)  13 (100)  11 (85)  13 (100)  13 (100) | 0  1 (20)  0  0  0  0  0  3 (23)  2 (15)  0  1 (8)  0  2 (15)  0  0 | 1 (20)  0  0  0  0  1 (20)  0  0  1 (8)  1 (8)  0  0  0  0  0 |

Legend: C. significant, clinically significant. Values in brackets indicate percentages.

Correlations between visual acuity/genotype and phenotype (neurodevelopmental, cognitive, adaptive and behavioral features)

Considering the cognitive level, we observed a statistically significant association between visual acuity and PIQ (*p*=0.001) and PSI (*p*=0.021). As regards adaptive functioning, statistical correlations were found between visual acuity and VABS-II total score (*p*=0.020), communication (*p*=0.020) and socialization (*p*=0.037) domains. No significant correlations were detected between visual acuity and emotional and behavioral difficulties at CBCL.

No significant correlations emerged between OCA1 and OCA2 subgroups according to the neurodevelopmental (motor milestones, p=0.06, language skills, p=0.1, and DQ at Griffiths-R, p=0.1), cognitive (FSIQ, p=0.5), adaptive (total score at VABS II, p=0.9), and behavioral (total score at CASD, p=0.5, CBCL total problems, p=0.6, internalizing p=0.3, and externalizing p=0.5) profiles.

**DISCUSSION**

OCA is a rare genetic disorder caused by the complete absence or reduction of biosynthesis of melanin in melanocytes resulting in mild to severe depigmentation of the hair, skin, and eyes [3]. Therefore, it is not surprising that research on subjects affected by OCA has been mainly focused on cutaneous and ocular signs and symptoms. To date, the neurodevelopmental profile has been poorly investigated, although several evidences support the hypothesis that brain may be involved in OCA: directly, due to the disruption of the mechanisms involved in the conversion of tyrosine in molecules essential for the retinal and visual network development [~~39~~ 40]; indirectly, as a consequence of congenital and often severe visual problems that could interfere with neurodevelopment, as documented in OA children [~~21~~ 22] and in other clinical populations [~~23; 40~~ 24; 41]. In the present study we aimed at detailing the neurological and neurovisual skills, cognitive level, adaptive functioning, and behavioral aspects of children with OCA, also exploring a possible correlation between the level of visual acuity deficit/genotype (OCA1 *vs* OCA2) and the clinical profile.

About half of the children (56%) presented an impairment in global neurodevelopment at an early stage. This percentage seems to be higher than that reported in pediatric population-based studies (neurodevelopmental delay between 4.5% and 12%) [~~41~~ 42], and similar to that observed in children with Leber’s Congenital Amaurosis (45%), “other” congenital retinal dystrophies (33%), or congenital disorders of the peripheral visual system (38-61%) [~~42-43~~ 43-44]. However, these studies used different measures to evaluate the neurodevelopment so it is difficult to compare the data. All the developmental areas of our children were affected, specifically gross-motor, personal-social, communicative, hand-eye coordination, and performance domains. It has been hypothesized that an early and severe visual deficit may affect the acquisition of motor skills [~~44-45~~ 45-46], daily life and social abilities [~~46~~ 47], language [~~47~~ 48] and cognition [~~42~~ 43]. Children typically develop through the interaction with the environment, a process mainly mediated by vision. In absence of the “incentive” represented by the sight [49], children may have difficulties to build up a picture of the world [~~44~~ 45] and develop gross and fine motor skills, above all during the second 6 months of life, when the movements begin to be enhanced by voluntary patterns and relational aspects (exploration, inquiry, etc.). In this regard, our children presented a slight delay in achieving head control and sitting position, and a marked delay for the other developmental milestones, such as walking, reached at an average age of 16 months. The development of prehension may be also impaired since the hands of the visually impaired child remain at shoulder height for a longer time [~~44~~ 45] without the natural behavior of environmental exploration. Furthermore, hand-eye coordination could be achieved later due to its dependence of hearing cue [~~49-50~~ 50-51]. Our children appropriately acquired the early fine motor milestones, later showing difficulties in hand-eye coordination, probably because the visual acuity deficit was not so severe as to limit the explorative-tactile function of their hands. The peculiar perceptual experience due to visual deficits may also limit the language development [~~47~~ 48], with difficulties in the construction of lexical semantics, which implies the construction of a relationship between language and extra-linguistic reality. The attribution of lexical labels to familiar people and objects needs the possibility of the child to look at what adult is looking at by following the direction of his gaze and thus associate signifier with signified. Therefore, the impairment in referential looking slows down lexical acquisition [~~51-52~~ 52-53]. In line with literature, 38% of our children presented a delay in word production and/or vocabulary expansion evolving in two cases in receptive and expressive language disorder. By contrast, in the general population approximately 15% of children have slow onset and progression of expressive language [~~53-54~~ 54-55] and 5–7% present a developmental language disorder [~~55-56~~ 56-57]. Vision also exerts a crucial role in the socio-relational skills development: eye contact represents the earliest mediator of mother-infant communication [~~57~~ 58] and thus, a visual impairment may lead to a distortion of this relationship resulting in personal-social difficulties as observed in our sample and in literature [~~57~~ 58]. Finally, vision is considered the most important sense for the construction of sensorimotor intelligence [~~58~~ 59]: through vision the child acquires the most important cognitive milestones such as the awareness of physical causality, spatial relations, and object permanence. An early-onset visual deficit may determine difficulties in the cognitive processing of reality. These data may explain why our children showed delayed score in performance and global domains at GMDS-R.

At the time of direct evaluation, all the children presented a normal neurological examination except for receptive and expressive language disorder (2 cases) and for a variable combination of visual signs and symptoms, in particular refractive errors, iris translucency, hypopigmentation of the retina, foveal hypoplasia, photophobia, abnormal head position, strabismus, nystagmus, unstable fixation, discontinued smooth pursuit, saccades dysfunctions, reduced visual acuity (moderate visual impairment/partial blindness), altered contrast sensitivity, and visual field limitations. Literature data report that these ocular, oculomotor, and basic visual functions manifestations are shared across all types of OCA [60].

We observed that the delayed score in DQ at GMDS-R do not evolve into an intellectual disability. The only child with delayed scores at FSIQ presented a PIQ within normal range and a severe language disorder that can justify the low score at verbal and total scale. Literature data on cognitive skills in subjects suffered from peripheral visual deficit are dated and scarce, reporting different results according to etiopathogenesis: studies conducted on albino subjects do not detect intellectual deficits [~~15-17~~ 16-18] and, thus, support our observations, while cognitive disability was found in patients with Leber’s Congenital Amaurosis (50%), probably for an association with central nervous system (CNS) anomalies (such as microgyria, polygyria, hypoplasia of the cerebellar vermis, ventricular dilatation) [61]. In presence of a visual impairment, sound and touch can guide motor experience and exploration of the surrounding environment. The “reach and touch on sound” stage, i.e., the ability to reach out for and grasp an object presented exclusively through the sound, constitutes a topical moment in the development of visually impaired children, allowing them to access to the world of mental representation [~~44; 48~~ 45; 49]. Literature indicates that by the age of 36 months, most children with visual impairment fully acquire the reach on sound function, considered a condition of, and a catalyst to, all the subsequent achievements [~~44~~ 45]. In this regard, we hypothesize that the achievement of the reach on sound phase in our sample may have organized not only motor, but also cognitive and behavioral experience, determining a normal developmental trajectory. Furthermore, our data are in line with literature, reporting normal cognitive quotients in albino subjects [~~15-17~~ 16-18]. The level of visual acuity seemed to positively correlate with PIQ and PSI, probably because these scales may be more dependent from visual input.

Few subjects of our sample reported difficulties in adaptive skills according to the VABS-II interview. No data on adaptive functioning of individuals affected by OCA are available, but studies on visually impaired subjects documented inadequate skills in the majority of cases [~~61-62~~ 62-63]. This difference may be explained by the characteristics of sample in terms of 1) level of visual acuity (predominantly blindness in the above cited studies *vs* predominantly low vision in ours), 2) timing of visual deficit onset (different age *vs* congenital) and 3) aetiology (multiple causes *vs* OCA). As described by other authors [~~63-65~~ 64-66], we found that the level of visual acuity seemed to influence the VABS-II total score, as well as the communicative and social functioning domains: subjects with marked visual impairment have greater adaptive difficulties. Different factors may contribute to this positive relation. First, children with a basic level of vision may have a protection to social communicative development compared to those with profound visual impairment [~~64~~ 65]. In fact, vision could be considered as a mediator of verbal language development (as described above) as well as of non-verbal pragmatic behaviors of social interactions (such as eye contact, recognition of facial expression, and visual imitation) [~~64; 66~~ 65; 67]. Second, out of school activities such as socializing, dancing, playing non-team sports, going to movies etc., depends on sensory input and, thus, impairments may lead to lower performance in daily life skills and socialization [~~46; 61~~ 47; 62]. Furthermore, parents tend to be overprotective with their child, especially if blind, limiting them in coping with tasks by themselves [~~61; 63~~ 62; 64].

According to the CBCL, 33% of our children reached the clinical or subclinical range for internalizing problems, percentage that is higher than that observed in healthy pediatric population (anxiety disorders: 6.5%, depressive disorder: 2.6%) [~~67~~ 68]. Our findings mirrors previous studies that reported higher risk of emotionally reactive, anxiety, avoidant and withdrawal behaviours in peripheral visually impaired children (internalizing problems: 22.7%) [~~68~~ 69], although the exact mechanisms of this heightened risk are still unclear. It has been suggested that a visual reduction and the resulting limited sensory experiences may have a negative impact on emotional-behavioural self-regulation [~~69-70~~ 70-71]. More than half of our children (67%) showed one or more autism-like features. This prevalence is similar to that reported in children with optic nerve hypoplasia and septo-optic dysplasia (58%) [~~71~~ 72] and higher compared to congenital disorders of the peripheral visual system (20%) [~~37~~ 38], and pediatric population (12.5%) [~~72~~ 73]. However, none of our patients reached the cut-off criteria at CASD questionnaire for an ASD. The presence of a diagnosis of ASD in visually impaired children greatly varies among studies, and has been reported in 31% of subjects with optic nerve hypoplasia and septo-optic dysplasia [~~71~~ 72], 8-12% of children with congenital disorders of the peripheral visual system [~~37;73~~ 38; 74], and in 1% of general population [~~74~~ 75]. These differences may be explained by methodological heterogeneity (different instruments for evaluating autistic-related behaviours) and by the characteristics of samples (all children with albinism in our cohort and mixed form of peripheral visual impairment in the other studies). Given the occurrence of autistic-like features in visually impaired children, we hypothesis that these traits may reflect a blind-specific developmental problem in the acquisition of socio-cognitive abilities rather than a specific ASD [~~21;37;75~~ 22; 38; 76].

Finally, we did not find a specific neurodevelopmental, cognitive, adaptive, and behavioral profile according to the genetic defect (OCA1 *vs* OCA2). The absence of significant genotype-phenotype correlations has been also reported in a recent study conducted by Dumitrescu and colleagues (2021) on visual features in a cohort of patients with OCA [~~76~~ 77].

In conclusion, our findings suggest that children with OCA may present an early neurodevelopmental delay that tend to be solved with age. The adaptive functioning seemed to be spared but the level of visual acuity may influence the communicative and social abilities as well as the global adaptive behavior. Subjects may show risks for emotional-behavioral problems, as well as autistic-like features that need to be correctly judged to avoid an improper diagnosis [~~37;75~~ 38; 76]. We are aware that it is difficult to compare data due to differences in methodologies among studies, but the neurocognitive difficulties found in our sample are similar to those of children with peripheral visual impairment. Furthermore, it has been demonstrated the presence of CNS anomalies in albino subjects, such as an increase in cortical thickness of the occipital cortex similar to that found in peripherally blind subjects, suggesting an association between degraded visual input and changes in brain morphology [~~77~~ 78]. Therefore, we hypothesize that brain is particularly vulnerable to the effects of visual impairment. Nonetheless, alterations of the genes involved in albinism may play a role in the neurodevelopmental problems, since it has been documented their expression also in CNS [~~78-79~~ 79-80], but their role has not yet been clearly understood. Although it has been described that the association between OCA and neurodevelopmental delay might indicate the presence of Prader-Willi (PWS) or Angelman syndrome (AS) [~~80~~ 81], due to the adjacency of *OCA2* and *UBE3A* gene on chromosome 15, we exclude that neurocognitive profile observed in our children may be related to these conditions, because we found: 1) uneventful perinatal period (feeding problems and failure to thrive is reported in PWS); 2) mild motor, language and neurodevelopmental delay (“severe” in AS); 3) absence of intellectual disability (one of the hallmarks of AS and PWS); 4) no dysmorphic features and/or neurological signs and symptoms (such as hypotonia reported in PWS or microcephaly and movement or balance disorders in AS) [~~81-82~~ 82-83]. The neurocognitive signs observed in our cohort underline the importance of an early neuropsychiatric evaluation to identify those children with neurodevelopmental/psychological difficulties and to adopt habilitative interventions.

The small sample size and the heterogeneity regarding age are the two main limitations of the present study that do not allow the generalization of the findings. Although OCA is a rare genetic disease, future studies should be conducted in a larger cohort of participants to better characterize these aspects.

**Declarations**

**Funding**

The authors declare that no funds, grants, or other support were received during the preparation of this manuscript.

**Conflict of interest**

The authors declare no competing interests.

**Author Contributions**

JG, EF designed the study. JG, EL, LD collected data and drafted the manuscript. EL performed the statistical analysis. JG, AR enrolled the patients. JG, ND performed the visual evaluation, VS the cognitive evaluation and LM the genetic analyses. EF supervised the study. All authors read and approved the final manuscript.

**Acknowledgments**

We thank children affected by oculocutaneous albinism and their care providers for participating and Albinit APS as well. The authors are grateful to Anna Alessandrini, Alice Bertoletti, Nicole D’Adda, Alessandra Franzoni, and Melissa Marras for their valuable help in evaluating the children included in this study.

**Ethical approval and informed consent**

The study was approved by the Ethical Committee of ASST Spedali Civili in Brescia, Italy (NP 5523). Written informed consent was obtained from parents/caregivers of the children.

**REFERENCES**

1. David CV (2013) Oculocutaneous albinism. Cutis, 91(5), E1–E4.
2. Ma EZ, Zhou AE, Hoegler KM, Khachemoune A (2022) Oculocutaneous albinism: epidemiology, genetics, skin manifestation, and psychosocial issues. Archives of dermatological research, 10.1007/s00403-022-02335-1. Advance online publication. https://doi.org/10.1007/s00403-022-02335-1
3. Marçon CR, Maia M (2019) Albinism: epidemiology, genetics, cutaneous characterization, psychosocial factors. Anais brasileiros de dermatologia, 94(5), 503–520. https://doi.org/10.1016/j.abd.2019.09.023
4. Grønskov K, Ek J, Brondum-Nielsen K (2007) Oculocutaneous albinism. Orphanet journal of rare diseases, 2, 43. https://doi.org/10.1186/1750-1172-2-43
5. Kirkwood BJ (2009) Albinism and its implications with vision. Insight (American Society of Ophthalmic Registered Nurses), 34(2), 13–16.
6. Kruijt CC, de Wit GC, Bergen AA, Florijn RJ, Schalij-Delfos NE, van Genderen MM (2018) The Phenotypic Spectrum of Albinism. Ophthalmology, 125(12), 1953–1960. https://doi.org/10.1016/j.ophtha.2018.08.003
7. Oetting WS, King RA (1999) Molecular basis of albinism: mutations and polymorphisms of pigmentation genes associated with albinism. Human mutation, 13(2), 99–115. https://doi.org/10.1002/(SICI)1098-1004(1999)13:2<99::AID-HUMU2>3.0.CO;2-C
8. Wei AH, Zang DJ, Zhang Z, Liu XZ, He X, Yang L et al (2013) Exome sequencing identifies SLC24A5 as a candidate gene for nonsyndromic oculocutaneous albinism. The Journal of investigative dermatology, 133(7), 1834–1840. https://doi.org/10.1038/jid.2013.49
9. Grønskov K, Dooley CM, Østergaard E, Kelsh RN, Hansen L, Levesque MP et al (2013) Mutations in c10orf11, a melanocyte-differentiation gene, cause autosomal-recessive albinism. American journal of human genetics, 92(3), 415–421. <https://doi.org/10.1016/j.ajhg.2013.01.006>
10. Pennamen P, Tingaud-Sequeira A, Gazova I, Keighren M, McKie L, Marlin S, et al (2021) Dopachrome tautomerase variants in patients with oculocutaneous albinism. Genetics in medicine : official journal of the American College of Medical Genetics, 23(3), 479–487.
11. Kausar T, Bhatti MA, Ali M, Shaikh RS, Ahmed ZM (2013) OCA5, a novel locus for non-syndromic oculocutaneous albinism, maps to chromosome 4q24. Clinical genetics, 84(1), 91–93. https://doi.org/10.1111/cge.12019
12. Merideth MA, Introne WJ, Wang JA, O'Brien KJ, Huizing M, Gochuico BR (2020) Genetic variants associated with Hermansky-Pudlak syndrome. Platelets, 31(4), 544–547. https://doi.org/10.1080/09537104.2019.1663810
13. Lozano ML, Rivera J, Sánchez-Guiu I, Vicente V (2014) Towards the targeted management of Chediak-Higashi syndrome. Orphanet journal of rare diseases, 9, 132. https://doi.org/10.1186/s13023-014-0132-6
14. Kutzbach B, Summers CG, Holleschau AM, King RA, MacDonald JT (2007) The prevalence of attention-deficit/hyperactivity disorder among persons with albinism. Journal of child neurology, 22(12), 1342–1347. https://doi.org/10.1177/0883073807307078
15. Kutzbach BR, Summers CG, Holleschau AM, MacDonald JT (2008) Neurodevelopment in children with albinism. Ophthalmology, 115(10), 1805–1808.e18082. https://doi.org/10.1016/j.ophtha.2008.03.006
16. Cole GF, Conn P, Jones RB, Wallace J, Moore VR (1987) Cognitive functioning in albino children. Developmental medicine and child neurology, 29(5), 659–665. https://doi.org/10.1111/j.1469-8749.1987.tb08508.x
17. Fulcher T, O'Keefe M, Bowell R, Lanigan B, Burke T, Carr A et al (1995) Intellectual and educational attainment in albinism. Journal of pediatric ophthalmology and strabismus, 32(6), 368–372. https://doi.org/10.3928/0191-3913-19951101-09
18. Keeffe JE (1990) Assessment and educational implications of albinism. Ophthalmic paediatrics and genetics, 11(3), 215–224. https://doi.org/10.3109/13816819009020982
19. Rogawski MA, Funderburk SJ, Cederbaum SD (1978) Oculocutaneous albinism and mental disorder. A report of two autistic boys. Human heredity, 28(2), 81–85. https://doi.org/10.1159/000152946
20. Delong R (2007) GABA(A) receptor alpha5 subunit as a candidate gene for autism and bipolar disorder: a proposed endophenotype with parent-of-origin and gain-of-function features, with or without oculocutaneous albinism. Autism: the international journal of research and practice, 11(2), 135–147. https://doi.org/10.1177/1362361307075705
21. Hesapcioglu S (2013) Oculocutaneous albinism and autism: a case report and review of literature. Düşünen Adam-Psikiyatri ve Nörolojik Bilimler Dergisi, 26(2), 215-218.
22. Galli J, Loi E, Morandi A, Scaglioni V, Rossi A, Molinaro A et al (2022) Neurodevelopmental Profile in Children Affected by Ocular Albinism. Neuropediatrics, 53(1), 7–14. https://doi.org/10.1055/s-0041-1732430
23. Green E, Stroud L, Bloomfield S, Cronje J, Foxcroft C, Hurter K (2017) Griffiths III. Griffiths Scales of Child Development, Third Edn, eds S. Lanfranchi, M. Rea, R. Vianello, and R. Ferri (Firenze: Hogrefe). Edizione Italiana a cura.
24. Fazzi E, Micheletti S, Calza S, Merabet L, Rossi A, Galli J et al (2021) Early visual training and environmental adaptation for infants with visual impairment. Developmental medicine and child neurology, 63(10), 1180–1193. https://doi.org/10.1111/dmcn.14865
25. Galli J, Loi E, Molinaro A, Calza S, Franzoni A, Micheletti S et al (2022) Age-Related Effects on the Spectrum of Cerebral Visual Impairment in Children With Cerebral Palsy. Frontiers in human neuroscience, 16, 750464. https://doi.org/10.3389/fnhum.2022.750464
26. Galli J, Loi E, Strobio C, Micheletti S, Martelli P, Merabet LB et al (2022) Neurovisual profile in children affected by Angelman syndrome. Brain & development, S0387-7604(22)00171-1. Advance online publication. https://doi.org/10.1016/j.braindev.2022.10.003
27. Teller DY, McDonald MA, Preston K, Sebris SL, Dobson V (1986) Assessment of visual acuity in infants and children: the acuity card procedure. Developmental medicine and child neurology, 28(6), 779–789. https://doi.org/10.1111/j.1469-8749.1986.tb03932.x
28. Hyvärinen L, Näsänen R, Laurinen P (1980) New visual acuity test for pre-school children. Acta ophthalmologica, 58(4), 507–511. https://doi.org/10.1111/j.1755-3768.1980.tb08291.x
29. Donahue SP, Baker CN, Committee on Practice and Ambulatory Medicine, American Academy of Pediatrics, Section on Ophthalmology, American Academy of Pediatrics, American Association of Certified Orthoptists, American Association for Pediatric Ophthalmology and Strabismus, American Academy of Ophthalmology (2016) Procedures for the Evaluation of the Visual System by Pediatricians. Pediatrics, 137(1), 10.1542/peds.2015-3597. https://doi.org/10.1542/peds.2015-3597
30. World Health Organization [WHO] (2021). ICD-10: International Statistical Classification of Diseases and Related Health Problems: Tenth Revision, 2nd Edn. Geneva: World Health Organization.
31. van Hof-van Duin J, Heersema DJ, Groenendaal F, Baerts W, Fetter WP (1992) Visual field and grating acuity development in low-risk preterm infants during the first 2 1/2 years after term. Behavioural brain research, 49(1), 115–122. https://doi.org/10.1016/s0166-4328(05)80201-3
32. Wechsler D (2002) WPPSI: Technical and Interpretative Manual. San Antonio, TX: The Psychological Corporation.
33. Wechsler D (2003) Wechsler Intelligence Scale for Children, 4th Edn. San Antonio, TX: Harcourt Assessment.
34. Sparrow SS, Cicchetti DV, Balla DA (2016) Vineland Adaptive Behavior Scales-II—2nd ed. Survey Interview Firenze: Giunti Psychometrics.
35. Achenbach TM, Rescorla LA (2000) Manual for the ASEBA preschool forms and profiles: An Integrated System of Multi-Informant Assessment. Burlington, VT: University of Vermont, Department of Psychiatry.
36. Frigerio A, Cozzi P, Pastore V, Molteni M, Borgatti R, Montirosso R (2006) La valutazione dei problemi emotivo comportamentali in un campione italiano di bambini in eta prescolare attraverso la Child Behavior Checklist e il Caregiver Teacher Report Form. Infanzia e adolescenza.
37. Achenbach TM, Rescorla LA (2001) Manual for the ASEBA School-Age Forms & Profiles: Child Behavior Checklist for Ages 6-18, Teacher’s Report Form, Youth Self-Report: An Integrated System of Multi-Informant Assessment. Burlington: University of Vermont, Research Center for Children Youth & Families.
38. Fazzi E, Micheletti S, Galli J, Rossi A, Gitti F, Molinaro A (2019) Autism in Children With Cerebral and Peripheral Visual Impairment: Fact or Artifact?. Seminars in pediatric neurology, 31, 57–67. https://doi.org/10.1016/j.spen.2019.05.008
39. Mayes S (2012) Checklist for Autism Spectrum Disorder. Wood Dale, IL: Stoelting.
40. Lee H, Purohit R, Sheth V, Maconachie G, Tu Z, Thomas MG et al (2023) Retinal Development in Infants and Young Children With Albinism: Evidence for Plasticity in Early Childhood. American journal of ophthalmology, 245, 202–211. https://doi.org/10.1016/j.ajo.2022.08.028
41. Purpura G, Bacci GM, Bargagna S, Cioni G, Caputo R, Tinelli F (2019) Visual assessment in Down Syndrome: The relevance of early visual functions. Early human development, 131, 21–28. https://doi.org/10.1016/j.earlhumdev.2019.01.020
42. Villagomez AN, Muñoz FM, Peterson RL, Colbert AM, Gladstone M, MacDonald B, et al (2019) Brighton Collaboration Neurodevelopmental Delay Working Group. Neurodevelopmental delay: Case definition & guidelines for data collection, analysis, and presentation of immunization safety data. Vaccine, 37(52), 7623–7641. https://doi.org/10.1016/j.vaccine.2019.05.027
43. Dale N, Sonksen P (2002) Developmental outcome, including setback, in young children with severe visual impairment. Developmental medicine and child neurology, 44(9), 613–622. https://doi.org/10.1017/s0012162201002651
44. Black MM, and Sonksen PM (1992). Congenital retinal dystrophies: a study of early cognitive and visual development. Archives of disease in childhood, 67(3), 262–265. https://doi.org/10.1136/adc.67.3.262
45. Elisa F, Josée L, Oreste FG, Claudia A, Antonella L, Sabrina S et al (2002) Gross motor development and reach on sound as critical tools for the development of the blind child. Brain & development, 24(5), 269–275. https://doi.org/10.1016/s0387-7604(02)00021-9
46. Hallemans A, Ortibus E, Truijen S, Meire F (2011) Development of independent locomotion in children with a severe visual impairment. Research in developmental disabilities, 32(6), 2069–2074. https://doi.org/10.1016/j.ridd.2011.08.017
47. Engel-Yeger B, Hamed-Daher S (2013) Comparing participation in out of school activities between children with visual impairments, children with hearing impairments and typical peers. Research in developmental disabilities, 34(10), 3124–3132. https://doi.org/10.1016/j.ridd.2013.05.049
48. Mosca R, Kritzinger A, van der Linde J (2015) Language and communication development in preschool children with visual impairment: A systematic review. The South African journal of communication disorders = Die Suid-Afrikaanse tydskrif vir Kommunikasieafwykings, 62(1), e1–e10. https://doi.org/10.4102/sajcd.v62i1.119
49. Fraiberg S (1977) Insights from the blind: Comparative studies of blind and sighted infants. New York: Basic Books.
50. Perez-Pereira M, Conti-Ramsden G (1999) Social interaction and language development in blind children. Hove: Psychology press.
51. Clifton RK, Rochat P, Litovsky RY, Perris EE (1991) Object representation guides infants' reaching in the dark. Journal of experimental psychology. Human perception and performance, 17(2), 323–329. https://doi.org/10.1037//0096-1523.17.2.323
52. Peltzer-Karpf A (2012) The dynamic landscape of exceptional language development. Strabismus, 20(2), 69–73. https://doi.org/10.3109/09273972.2012.680236
53. Glass P (2002) Development of the visual system and implications for early intervention. Infants and Young Children, 15(1), 1–10. http://dx.doi.org/10.1097/00001163-200207000-00003
54. Collisson BA, Graham SA, Preston JL, Rose MS, McDonald S, Tough S (2016) Risk and protective factors for late talking: An epidemiologic investigation. The Journal of pediatrics, 172, 168–174. https://doi.org/10.1016/j.jpeds.2016.02.020
55. Horwitz SM, Irwin JR, Briggs-Gowan MJ, Bosson Heenan JM, Mendoza J, Carter AS (2003) Language delay in a community cohort of young children. Journal of the American Academy of Child and Adolescent Psychiatry, 42(8), 932–940. https://doi.org/10.1097/01.CHI.0000046889.2726
56. Norbury CF, Gooch D, Wray C, Baird G, Charman T, Simonoff E (2016) The impact of nonverbal ability on prevalence and clinical presentation of language disorder: Evidence from a population study. Journal of Child Psychology and Psychiatry, 57, 1247–1257. https://doi.org/10.1111/jcpp.12573
57. Law J, Boyle J, Harris F, Harkness A, Nye C (2000) Prevalence and natural history of primary speech and language delay: findings from a systematic review of the literature. International journal of language & communication disorders, 35(2), 165–188. https://doi.org/10.1080/136828200247133
58. Trevarthen C, Aitken KJ (2001) Infant intersubjectivity: research, theory, and clinical applications. Journal of child psychology and psychiatry, and allied disciplines, 42(1), 3–48.
59. Piaget J, Cook MT (1952) The origins of intelligence in children.
60. Kinnear PE, Jay B, Witkop Jr CJ (1985) Albinism. Survey of ophthalmology, 30(2), 75-101.
61. Steinberg A, Ronen S, Zlotogorski Z, Silverston BZ, Hirsch I, Nawratzki I (1992) Central nervous system involvement in Leber congenital amaurosis. Journal of pediatric ophthalmology and strabismus, 29(4), 224–227. https://doi.org/10.3928/0191-3913-19920701-09
62. Papadopoulos K, Metsiou K, Agaliotis I (2011) Adaptive behavior of children and adolescents with visual impairments. Research in developmental disabilities, 32(3), 1086–1096. https://doi.org/10.1016/j.ridd.2011.01.021
63. Metsiou K, Papadopoulos K, Agaliotis I (2011) Adaptive behavior of primary school students with visual impairments: the impact of educational settings. Research in developmental disabilities, 32(6), 2340–2345. https://doi.org/10.1016/j.ridd.2011.07.030
64. Bathelt J, de Haan M, Dale NJ (2019) Adaptive behaviour and quality of life in school-age children with congenital visual disorders and different levels of visual impairment. Research in developmental disabilities, 85, 154–162. https://doi.org/10.1016/j.ridd.2018.12.003
65. Dale NJ, Tadić V, Sonksen P (2014) Social communicative variation in 1-3-year-olds with severe visual impairment. Child: care, health and development, 40(2), 158–164. https://doi.org/10.1111/cch.12065
66. Elsman EBM, Koel M, van Nispen RMA, van Rens GHMB (2021) Quality of Life and Participation of Children With Visual Impairment: Comparison With Population Reference Scores. Investigative ophthalmology & visual science, 62(7), 14. https://doi.org/10.1167/iovs.62.7.14
67. Tadić V, Pring L, Dale N (2010) Are language and social communication intact in children with congenital visual impairment at school age?. Journal of child psychology and psychiatry, and allied disciplines, 51(6), 696–705. https://doi.org/10.1111/j.1469-7610.2009.02200.x
68. Polanczyk GV, Salum GA, Sugaya LS, Caye A, Rohde LA (2015) Annual research review: A meta-analysis of the worldwide prevalence of mental disorders in children and adolescents. Journal of child psychology and psychiatry, and allied disciplines, 56(3), 345–365. https://doi.org/10.1111/jcpp.12381
69. O'Reilly MA, Bathelt J, Sakkalou E, Sakki H, Salt A, Dale N et al (2017) Frontal EEG asymmetry and later behavior vulnerability in infants with congenital visual impairment. Clinical neurophysiology : official journal of the International Federation of Clinical Neurophysiology, 128(11), 2191–2199. https://doi.org/10.1016/j.clinph.2017.08.016
70. Alon L, Cohen Ophir M, Cohen A, Tirosh E (2010) Regulation disorders among children with visual impairment a controlled study. Journal of Developmental and Physical Disabilities, 22(1), 57-64. <https://doi.org/10.1007/s10882-009-9169-1>
71. Chennaz L, Valente D, Baltenneck N, Baudouin JY, Gentaz E (2022) Emotion regulation in blind and visually impaired children aged 3 to 12 years assessed by a parental questionnaire. Acta psychologica, 225, 103553. https://doi.org/10.1016/j.actpsy.2022.103553
72. Parr JR, Dale NJ, Shaffer LM, Salt AT (2010) Social communication difficulties and autism spectrum disorder in young children with optic nerve hypoplasia and/or septo-optic dysplasia. Developmental medicine and child neurology, 52(10), 917–921. <https://doi.org/10.1111/j.1469-8749.2010.03664.x>
73. Lundström S, Chang Z, Råstam M, Gillberg C, Larsson H, Anckarsäter H, et al (2012) Autism spectrum disorders and autistic like traits: similar etiology in the extreme end and the normal variation. Archives of general psychiatry, 69(1), 46–52. https://doi.org/10.1001/archgenpsychiatry.2011.144
74. Bathelt J, Dale N, de Haan M (2017) Event-related potential response to auditory social stimuli, parent-reported social communicative deficits and autism risk in school-aged children with congenital visual impairment. Developmental cognitive neuroscience, 27, 10–18. https://doi.org/10.1016/j.dcn.2017.07.003
75. Zeidan J, Fombonne E, Scorah J, Ibrahim A, Durkin MS, Saxena S, et al (2022) Global prevalence of autism: A systematic review update. Autism research: official journal of the International Society for Autism Research, 15(5), 778–790. https://doi.org/10.1002/aur.2696
76. Molinaro A, Micheletti S, Rossi A, Gitti F, Galli J, Merabet LB, et al (2020) Autistic-Like Features in Visually Impaired Children: A Review of Literature and Directions for Future Research. Brain sciences, 10(8), 507. <https://doi.org/10.3390/brainsci10080507>
77. Dumitrescu AV, Tran J, Pfeifer W, Bhattarai SV, Kemerley A, Dunn TV, et al (2021). Clinical albinism score, presence of nystagmus and optic nerves defects are correlated with visual outcome in patients with oculocutaneous albinism. Ophthalmic genetics, 42(5), 539–552. https://doi.org/10.1080/13816810.2021.1933544
78. Bridge H, von dem Hagen EA, Davies G, Chambers C, Gouws A, Hoffmann M, et al (2014) Changes in brain morphology in albinism reflect reduced visual acuity. Cortex; a journal devoted to the study of the nervous system and behavior, 56, 64–72. https://doi.org/10.1016/j.cortex.2012.08.010
79. Bakker R, Wagstaff EL, Kruijt CC, Emri E, van Karnebeek CDM, Hoffmann MB, et al (2022) The retinal pigmentation pathway in human albinism: Not so black and white. Progress in retinal and eye research, 91, 101091. https://doi.org/10.1016/j.preteyeres.2022.101091
80. Ginger RS, Askew SE, Ogborne RM, Wilson S, Ferdinando D, Dadd T, et al (2008) SLC24A5 encodes a trans-Golgi network protein with potassium-dependent sodium-calcium exchange activity that regulates human epidermal melanogenesis. The Journal of biological chemistry, 283(9), 5486–5495. https://doi.org/10.1074/jbc.M707521200
81. Saadeh R, Lisi EC, Batista DA, McIntosh I, Hoover-Fong JE (2007) Albinism and developmental delay: the need to test for 15q11-q13 deletion. Pediatric neurology, 37(4), 299–302. https://doi.org/10.1016/j.pediatrneurol.2007.06.024
82. Micheletti S, Palestra F, Martelli P, Accorsi P, Galli J, Giordano L, et al (2016) Neurodevelopmental profile in Angelman syndrome: more than low intelligence quotient. Italian journal of pediatrics, 42(1), 91. <https://doi.org/10.1186/s13052-016-0301-4>
83. Cassidy SB, Schwartz S, Miller JL, Driscoll DJ (2012) Prader-Willi syndrome. Genetics in medicine: official journal of the American College of Medical Genetics, 14(1), 10–26. <https://doi.org/10.1038/gim.0b013e31822bead0>
